# Genetic Variability of RXRB, PPARA, and PPARG in Wegener's Granulomatosis

**DOI:** 10.1155/2009/786781

**Published:** 2009-02-11

**Authors:** Stefan Wieczorek, Silvia Knaup, Wolfgang L. Gross, Jörg T. Epplen

**Affiliations:** ^1^Human Genetics Department, Ruhr University, 44801 Bochum, Germany; ^2^Department of Rheumatology, University Hospital Lübeck and Klinikum Bad Bramstedt, 24576 Bad Bramstedt, Germany

## Abstract

A major genomic region involved in Wegener's granulomatosis includes the gene for retinoid receptor beta (*RXRB*) which forms heterodimers with peroxisome proliferator-activated receptors (PPARs). It is unclear whether this association directly arises from the *RXRB* allele(s) or via a linked variation. In order to reveal any hitherto unknown and potentially disease-relevant variation of the *RXRB* gene, we have genotyped four tagging SNPs of this genomic region and have directly sequenced selected WG patients and controls representing disease-associated haplotypes. Additionally, we have genotyped 2 SNPs each in the genes for PPAR*α* and PPAR*γ* (*PPARA* and *PPARG*). Hence, we confirmed the strong association of the *RXRB* locus with WG but could not reveal any novel variation in *RXRB*. None of the *PPARA* and *PPARG* SNPs showed association with WG. Moreover, no epistatic effect was seen between *RXRB* and *PPARA*/*PPARG* alleles. These results do not support an etiopathological role of PPAR in WG. Analyses of further genes functionally linked to RXRB may provide additional data useful to evaluate the *RXRB* association found in WG.

## 1. Introduction

Wegener's granulomatosis (WG) is a form of small vessel vasculitis belonging to the group antineutrophil cytoplasmic antibody- (ANCA-) associated vasculitides (AAV). WG usually presents with granulomatous lesions of the upper respiratory tract and often develops into generalized vasculitis with multiple organ involvement in later disease stages. In WG ANCA typically recognize proteinase 3, delineating this diagnostic entity from other AAV forms like Churg Strauss syndrome or microscopic polyangiitis, in which ANCA are usually directed against myeloperoxidase (reviewed in [1]). Current concepts of its etiopathology are incomplete, but WG is accounted to the large and heterogenous group of complex disorders arise from a mostly elusive interplay of environmental and genetic factors.

A major genomic locus for WG was identified on chromosome 6p21.3, including (among others) the genes for *HLA-DPB1, RXRB*, and *RING1* [2]. The linkage disequilibrium (LD) pattern of this genomic region is complex with larger blocks of strong LD alternating with regions of high recombination rates. There is evidence that the association of this locus with the disorder may arise from more than one variation, making 6p21.3 a quantitative trait locus for WG. While a strong association is demonstrable with the HLA-DPB1*0104 allele, a (partly) independent association was detected for the defined region including potential WG candidate genes *RING1* and *RXRB* [3]. In addition certain alleles of *RXRB*, the gene encoding retinoid X receptor beta, are highly significantly associated with WG susceptibility [4].

Peroxisome proliferator-activated receptors (PPAR) are group of nuclear receptors mediating the effects of peroxisome proliferators on gene transcription. Binding of PPAR to regulatory DNA sequences requires the heterodimerization with RXR. PPAR mediated effects are complex and include modulation of inflammatory processes. PPAR*α* is a ligand for leukotriene B4, and PPAR*α* deficient mice show a prolonged response to inflammatory stimuli [5]. PPAR*α* also appears to be involved in downregulation of the activity of cyclooxigenase 2 (COX2) and nuclear factor kappa-b (NF*κ*B). Likewise, PPAR*γ* exhibits anti-inflammatory properties. It is upregulated in activated macrophages and inhibits the expression, for example, of the inducible nitric oxide synthase [6].

Genetic polymorphisms of PPAR have been investigated in numerous conditions, predominantly in metabolic disorders like type 2 diabetes or atherosclerosis [7–9]. There is evidence for accelerated atherosclerosis in WG patients [10], suggesting that both conditions may share some (genetic) risk factors.

Given the pleiotropic role of PPAR in the regulation of (vascular) inflammation, we have hypothesised that genetic predisposition to WG may arise from the interaction of certain genetic variations of RXRB and PPAR. We have, therefore, screened the *RXRB* gene for novel, potentially WG-specific variations. Moreover, we have genotyped two single nucleotide polymorphisms in each of the genes for PPAR*α* and PPAR*γ* in large panels of WG cases and healthy controls. Finally, we searched for epistatic effects of certain alleles of *RXRB* and *PPARA* or *PPARG*.

## 2. Patients and Methods

### 2.1. Subjects

All patients included in this study were diagnosed with WG according to the criteria of the *American College of Rheumatology* and the Chapel Hill consensus conference [11]. They were selected at the interdisciplinary vasculitis centre at the University of Luebeck/Rheumaklinik Bad Bramstedt. All patients were asked for their ancestry and reported German decent for at least two generations. Healthy German blood donors were used as controls and ancestry was evaluated equal to the patient group.

For the *RXRB* locus we genotyped the same patients (*n* = 282) and controls (*n* = 380) that were previously analyzed for the 6p21.3 locus [3]. In order to increase statistical power, the SNP in the PPAR genes were analyzed in expanded sample comprising 462 WG cases and 701 controls.

Ethical principles for medical research involving human subjects as defined in the *Declaration of Helsinki* have been followed. The study design was approved by the local ethics committee at the University of Luebeck, Germany (No. AZ 06-087).

### 2.2. Genotyping

**Figure 1:**
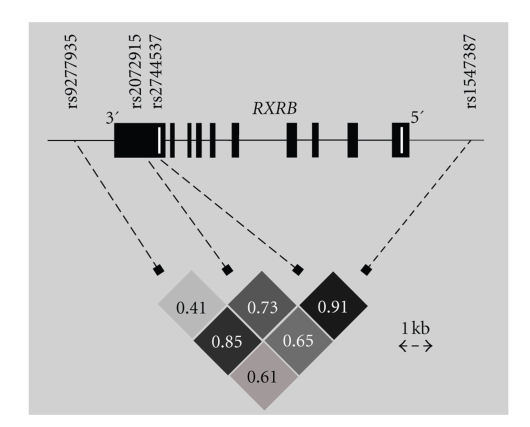
Location of the four tagging SNPs of the *RXRB* gene and LD structure. The LD is plotted as *D′* and intensity coded (darker shades corresponding to higher degree of LD). The *RXRB* gene is transcribed from centromer to telomer, that is, from right to left. *RXRB* exons are depicted as black boxes, and translation start and stop points are marked by white vertical lines.

Four types SNPs rs9277935, rs2072915, rs2744537, and rs1547387 (see Figure 1) were selected from the HapMap database to serve as tagSNPs for a region of 10 kb including the *RXRB* gene. With this selection all HapMap SNPs within this region are efficiently tagged (*r* ^2^ > 0.9). rs2072915 and rs2744537 are located within the 3′ untranslated region (UTR) of *RXRB,* while rs9277935 is located approximately 1 kb 3′ of the last *RXRB* exon. rs1547387 is located 5′ of *RXRB*, in exon 5 of the *SLC39A7* gene, in which it constitutes a silent SNP (Ser209Ser).

rs9277935, rs2072915, and rs2744537 were genotyped via PCR-RFLP techniques and a commercially available TaqMan genotyping assay (Applied Biosystems) was used for rs1547387.

For rs1800206 (Val162Leu) in *PPARA* also a commercially available TaqMan assay was used, while rs6008259 (3′ UTR of *PPARA*), rs1801282 (*PPARG*, Ala12Pro), and rs3856806 (*PPARG*, His449His) were genotyped using newly designed primers and probes (see Table 1).

### 2.3. Direct Sequencing of *RXRB*

The entire *RXRB* gene (all 10 exons and exon/intron boundaries) was directly sequenced in 5 WG patients typed homozygous for the associated *RXRB* haplotype (see below; primer sequences available on request). For comparison 5 healthy controls homozygous for the inversely associated (i.e., protective) haplotype were also sequenced.

### 2.4. Statistical Analysis

Genotypes were recorded in linkage format. Association for each single marker was tested by using chi square tests on contingency tables. A *P* value <.05 was considered significant. LD between each SNP of a respective locus, and haplotype block frequencies were calculated by using Haploview 4.1 [12].

Interaction between *RXRB* and *PPARA*/*PPARG* SNPs were calculated with GAIA (http://gump.qimr.edu.au/GAIA/gaia.html; [13]). This application uses a logistic regression model which tests for pairwise locus/locus interactions between genes. We applied an additive interaction model for each pair of SNPs testing the significance of the interaction model terms over and above any main effects.

## 3. Results and Discussion

The overall call rate for all 8 SNPs in patients and controls was 93.60%. None of the SNPs revealed significant deviation from Hardy-Weinberg equilibrium. Two of the *RXRB* SNPs (rs9277935 and rs2744537) showed highly significant association with WG (see Table 2). Accordingly, the GTTC haplotype (calculated from all four SNPs of this locus) was significantly more common in WG while the TTGC haplotype was overrepresented in controls (see Table 2). Based on the SNP data we have then selected 5 WG patients homozygous for the GTTC haplotype for direct sequencing of the *RXRB* gene. For comparison 5 control subjects homozygous for the TTGC haplotype were also analyzed. Yet, no novel sequence variation was detected, a finding which is consistent with previous results from Szyld et al. [4], who had screened the *RXRB* gene in WG patients without consideration of the haplotype structure of this locus. Results from the initial tagging SNP genotyping were confirmed in the ten included individuals for the two exonic tagging SNPs (rs2744537 and rs2072915). Two other exonic SNPs (rs1152296 in exon 10/5′UTR and rs6531 in exon 7) were identified and perfectly segregated with the previously identified risk/nonrisk haplotypes.

**Figure 2:**
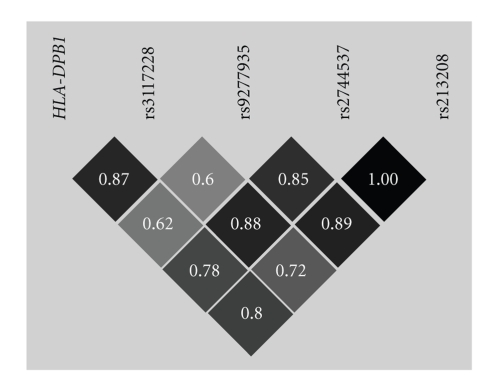
LD between WG associated variations of the 6p21.3 genomic region. *HLA-DPB1* is modelled as a biallelic marker with the associated HLA-DPB1*0401 as one allele and all other alleles joint into the other allelic category. LD is plotted as *D′* and intensity coded (darker shades corresponding to higher degree of LD). Both *RXRB* SNPs investigated in this study (rs9277935 and rs2744537) are in high LD with *HLA-DPB1* as well as with the markers analyzed by Heckmann et al. (2008): rs3117228 (3′ of *HLA-DPB1*) and rs213208 (intronic SNP in *RING1*, approx. 10 kb centromeric of *RXRB*).

Both associated *RXRB* SNPs (rs9277935 and rs2744537) are in strong LD with *HLA-DPB1* as well as with rs3117228 and rs213208 (see Figure 2) which had revealed the highest (and partly *HLA-DPB1* independent) association with WG in a previous study [3]. Moreover, according to the HapMap data rs2744537 is in strong LD with rs6531 (a synonymous SNP in exon 7 of *RXRB*) which was significantly associated with WG earlier [4]. As for the other associated SNPs in this genomic region, it is therefore hard to differentiate which of the SNPs (or even a yet unknown variation of this locus) constitutes the primary WG risk factor. A potential approach to overcome this problem is the analysis of factors related to the different proteins encoded in the 6p21.3 region. We have therefore investigated SNPs in the genes for PPAR*α* and PPAR*γ* which are functionally closely linked to RXRB.

The two SNPs in *PPARG* were in weak to moderate LD (*D′* = 0.64, *r^2^* = 0.40) while the two SNPs in *PPARA* were virtually unlinked (*D′* = 0.47, *r^2^* = 0.06). Therefore, no haplotype frequencies were calculated for these genes. Both SNPs in *PPARG* showed very similar allele frequencies in cases and controls not revealing any significant differences (see Table 2). The two SNPs in *PPARA* both revealed a decreased frequency of the minor allele but these differences did not reach significance level (see Table 2). No significant pairwise epistatic effect for any of the investigated SNPs, that is, neither between *PPARA* and *PPARG* nor between *PPARA* (or *PPARG*, resp.) and *RXRB* or *HLA-DPB1*. Taken together, we cannot provide evidence for a genetically based involvement of PPAR*α* and PPAR*γ* in the etiopathology of WG. Moreover, the strong WG association with the 6p21.3 locus is unlikely to be based on any coding variation of the *RXRB* gene. Future studies will therefore have to focus on regulatory elements of this area (e.g., *cis* acting elements or micro RNAs).

## 4. Conclusions

These results do not support a direct etiopathological role of RXRB and/or PPAR in WG. Analyses of further genes functionally linked to RXRB, for example, retinoic acid receptors or vitamin D receptors, may provide additional data useful to evaluate the *RXRB* association found in WG.

## Figures and Tables

**Table 1:** Oligonucleotides and restriction enzymes used for genotyping.

Gene	SNP	Forward (F) and reverse (R) primer (5′ > 3′)	Allele specific probes	Restriction enzyme	Fragment lengths
*RXRB*	rs9277935	F: TGCCCCTTGTAGGTCTCCAC ^(1)^	—	BtgI	T-Allel: 208 bp
	T/G	R: CCTCCCACTGTGCCCTAA			G-Allel: 34 + 174 bp
	rs2072915	F: ACATCTCCACCAGCCCCTTC	—	MboI	T-Allel: 325 bp
	T/A	R: GTCCTTCCCCCAGCACAAAG			A-Allel: 224 + 101 bp
	rs2744537	F: TCTTCAAGCTCATTGGTGAC	—	BsaI	T-Allel: 451 bp
	T/G	R: CCCATTTCCACTCTTCAGAT			G-Allel: 326 + 125 bp
	rs1547387	Information not provided by the manufacturer		—	—
	C/G				

*PPARA*	rs1800206	Information not provided by the manufacturer		—	—
	G/C				
	rs6008259	F: CCCCTGTGTCAACAAGATCCA	G: Fam-CTGTGTTGTCCCCAGCGACCC	—	—
	G/A	R: CTTGAATGGCACAGGGTACATC	A: YY-CCTGTGTTGTCCCCAACGACCC		

*PPARG*	rs1801282	F: TTATGGGTGAAACTCTGGGAGATT	G: Fam-TCCTATTGACGCAGAAAGCGATTCC	—	—
	G/C	R: TTGTGATATGTTTGCAGACAGTGTATC	C: YY-TCCTATTGACCCAGAAAGCGATTCCTT		
	rs3856806	F: CCAGAAAATGACAGACCTCAGACA	T: Fam-TCACGGAACATGTGCAGCTACTGC	—	—
	T/C	R: GGAGCGGGTGAAGACTCATG	C: YY-ATTGTCACGGAACACGTGCAGCTAC		

(1) A 17 mer nucleotide (GTAAAACGACGGCCAGT) was added 5′ to the forward primer increase fragment length differences between the two alleles, FAM: 6-carboxy-fluorescein; YY: Yakima Yellow.

**Table 2:** Allele and haplotype frequencies.

Gene	SNP/haplotype	Allele	Frequency in controls	Frequency WG patients	*P* value	OR (95% CI)
*RXRB*	rs9277935	G	0.76	0.89	6.65 × 10^−9^	2.55 (1.86–3.56)
		T	0.24	0.11		
	rs2072915	T	0.71	0.75	.157	1.20 (0.93–1.55)
		A	0.29	0.25		
	rs2744537	T	0.73	0.57	6.63 × 10^−9^	0.49 (0.39–0.63)
		G	0.27	0.43		
	rs1547387	C	0.87	0.85	.34	0.85 (0.62–1.18)
		G	0.13	0.15		

RXRB*	GTTC		0.24	0.40	1.15 × 10^−9^	2.11 (1.66–2.69)
	TTGC		0.19	0.07	9.73 × 10^−10^	0.32 (0.22–0.47)
	other		0.43	0.47	.157	1.18 (0.94–1.47)

*PPARA*	rs1800206	C	0.93	0.95	.076	1.40 (0.96–2.05)
		G	0.07	0.05		
	rs6008259	G	0.80	0.83	.069	0.81 (0.65–1.02)
		A	0.20	0.17		

*PPARG*	rs1801282	C	0.85	0.85	.74	0.96 (0.75–1.23)
		G	0.15	0.15		
	rs3856806	C	0.86	0.85	.70	0.95 (0.75–1.22)
		T	0.14	0.15		

OR: Odds ratio, CI: confidence interval, *haplotype frequencies calculated from all four *RXRB* SNPs.
